# Silencing of PGK1 Promotes Sensitivity to Paclitaxel Treatment by Upregulating XAF1-Mediated Apoptosis in Triple-Negative Breast Cancer

**DOI:** 10.3389/fonc.2021.535230

**Published:** 2021-03-04

**Authors:** Shanshan Sun, Hao Wu, Xiaohong Wu, Zilong You, Yang Jiang, Xiaoshuan Liang, Zhuo Chen, Ye Zhang, Wei Wei, Yongdong Jiang, Yanbo Chen, Yanni Song, Da Pang

**Affiliations:** ^1^Department of Breast Surgery, Harbin Medical University Cancer Hospital, Harbin, China; ^2^Sino-Russian Medical Research Center, Heilongjiang Academy of Medical Sciences, Harbin, China; ^3^Department of Anesthesiology, Harbin Medical University Cancer Hospital, Harbin, China; ^4^Department of Pathology, Harbin Medical University Cancer Hospital, Harbin, China; ^5^Department of Anesthesiology, Ningbo Medical Treatment Center Li Huili Hospital, Ningbo, China

**Keywords:** PGK1, triple-negative breast cancer, paclitaxel, sensitivity, chemotherapy

## Abstract

**Objective:** Triple negative breast cancer (TNBC) is known to have aggressive clinical course and a high risk of recurrence. Given the lack of effective targeted therapy options, paclitaxel-based chemotherapy is still the primary option for TNBC patients. However, patients who fail to achieve a complete response during neoadjuvant chemotherapy may be mainly due to sensitivity and resistance to chemotherapy. Thus, we concentrated the present research on the role of PGK1 in the sensitivity to paclitaxel treatment and the possible underlying mechanisms in TNBC.

**Methods:** After exposure to paclitaxel, a cell viability analysis was made to investigate the influence of PGK1 silencing on cell death. The effect of PGK1 on apoptosis induced by paclitaxel treatment was examined *in vitro* by flow cytometry cell apoptosis assays. Western blotting was performed to examine the impact of PGK1 on paclitaxel-induced apoptosis. The correlation of PGK1 with apoptosis-associated protein X-linked inhibitor of apoptosis (XIAP)-associated factor 1 (XAF1) was analyzed in 39 specimens by immunohistochemistry analysis.

**Results:** We observed that silencing PGK1 sensitized triple-negative breast cancer (TNBC) cell lines to paclitaxel treatment as a result of increased drug-induced apoptosis. Furthermore, mechanistic investigations suggested that XAF1 was increased in PGK1-knockdown cells along with the expression of the apoptotic proteins including cleaved caspase-3 and Bax. Immunohistochemistry analysis showed that PGK1 was negatively related to XAF1. Moreover, we found that downregulation of XAF1 reduced paclitaxel-induced apoptosis in PGK1-silenced triple-negative cell lines.

**Conclusion:** Our results identified PGK1 as a potential biomarker for the treatment of TNBC, and inhibition of PGK1 expression might represent a novel strategy to sensitize TNBC to paclitaxel treatment.

## Introduction

Currently, breast cancer is the most prevalent cancer in women worldwide. It accounted for 268,600 (30%) new cancer diagnoses and 41,760 (15%) cancer-related deaths in women in the United States (1). The same number of new cases is expected in women in China, but an increasing trend in mortality has been observed (2). Triple-negative breast cancer (TNBC) presents as a subtype express estrogen receptor (ER), progesterone receptor (PR) and human epidermal growth factor receptor 2 (Her-2) insufficiently. It accounts for 15–20% of whole breast cancers. It is known to have the most aggressive clinical course and a high risk of recurrence (3, 4). On account of deficiency of effective targeted therapy options, paclitaxel-based chemotherapy is still the primary option for TNBC patients. However, patients who do not achieve a complete response account for ~80% of TNBC cases, which may be mainly due to issues with sensitivity and resistance to chemotherapy (5). Recent evidence links hypoxia specifically with TNBC. Hypoxia might influence treatment outcomes negatively. It is associated with resistance to certain chemotherapeutic regimens (6–8). Therefore, considering the aggressive characteristic of TNBC, investigating hypoxia-related targets that mediate sensitivity to chemotherapy becomes a major strategy for improving TNBC treatment outcomes.

To survive the difficult condition of hypoxia, cells adopt characteristics of resistance necessarily, including enhancing glycolysis or overexpressing anti-apoptotic factors (9, 10). In hypoxic cells, glycolysis provides the main ATP content (11, 12); thus, glycolysis is a major energy metabolism mechanism of cancer cells. The energy released during this process is generated by the action some glycolytic enzymes (13, 14). Among the glycolytic enzymes, phosphoglycerate kinase-1 (PGK1) catalyzes the conversion of 1,3-diphosphoglycerate to 3-phosphoglycerate, generating one molecule of ATP (13). PGK1 has been recorded to have oncogenic roles and is associated with a poor prognosis in several cancers, such as prostate cancer (13), pancreatic cancer (15), gastric cancer (16, 17), liver cancer (18) and breast cancer (19, 20). In addition, increasing evidence has indicated that PGK1 participates in the hallmarks of cancer, including angiogenesis, DNA replication and repair in mammalian nuclei and tumor invasion and progression (17). PGK1 has also been reported to induce a multidrug-resistant (MDR) phenotype through an MDR-1-independent mechanism (21). Our previous study showed that PGK1 might be a predictor of poor survival and an immunohistochemical biomarker to predict the sensitivity to paclitaxel treatment in breast cancer (20). Inhibition of PGK1 promotes cell apoptosis in glioblastoma cells (22) and TNBC (23). Downregulation of PGK1 decreases ATP production and induces apoptosis (24). Depletion of ATP level, used in combination with chemotherapy has a variety of effects on cancer cells including inducing apoptosis in multidrug resistant cells (25). Since paclitaxel-based chemotherapy is still the primary option for TNBC patients, we concentrated the present research on the role of PGK1 in the sensitivity to paclitaxel treatment and the possible underlying mechanisms in TNBC.

Evasion of apoptosis is a major hallmark of cancer and has been linked with resistance to cytotoxic agents especially paclitaxel for the treatment of TNBC (26). Therefore, in this study, we found that silencing PGK1 sensitized MDA-MB-231 and MDA-MB-468 cell lines to paclitaxel treatment by activating the apoptosis pathway. To identify potential apoptosis-related molecular mechanisms of PGK1 in TNBC, RNA transcriptional sequence analysis was conducted to investigate the differential gene expression profiles. XAF1, associated with apoptosis, was chosen for further analysis. Furthermore, we evaluated whether knockdown of PGK1 increased the expression of XAF1. Moreover, we found that XAF1 might mediate PGK1-driven sensitivity to paclitaxel in TNBC cell lines. These novel findings have important implications for therapeutic strategies to induce paclitaxel sensitivity.

## Materials and Methods

### Patient Tissue Samples

This study purposed archival materials from the Department of Pathology in Harbin Medical University Cancer Hospital. The invasive breast cancer patients diagnosed in 2007 were involved. The exclusion criteria included patients with recurrent tumors, metastatic disease, bilateral tumors, or other previous tumors and patients received neoadjuvant treatment previously. All breast cancer patients were tested for ER, PR, and Her-2, which were assessed in paraffin-embedded, formalin-fixed tissues using antibodies against the ER, PR, and Her-2 proteins (Zhong Shan-Bio Co., Beijing, China), as we described previously (20). Thirty-nine TNBC tissue specimens were obtained. All protocols were reviewed and approved by the Ethical Committee of Harbin Medical University in Harbin, China. All patients gave informed consent for the diagnostic procedures and the proposed treatment (20).

### Cell Culture

MDA-MB-231 and MDA-MB-468 cell lines were acquired from Procell Life Science & Technology Co., Ltd. (Wuhan, Hubei, China) and were validated by analysis of STR (PowerPlex 18D system). The cell lines were cultured in high-glucose Dulbecco's modified Eagle's medium (DMEM) (Invitrogen, Carlsbad, CA) supplemented with 10% fetal bovine serum (Pan Biotech, Germany) at 37°C under an atmosphere of 5% CO_2_.

### Real-Time Quantitative PCR (Real-Time qPCR)

Total RNA was isolated from differentially treated cells using a Total RNA Kit I (Omega Biotech, Norcross, GA, USA) according to the manufacturers' instructions. The primers designed for PGK1 were as follows: forward, 5′AACCAGAGGATTAAGGCTGC3′ and reverse 5′GCCTACACAGTCCTTCAAGA3′. The primer sequences used to detect XAF1 were as follows: forward, 5′GCTCCACGAGTCCTACTGTG3′ and reverse, 5′GTTCACTGCGACAGACATCTC3′. β-actin was employed as internal reference. The primers of β-actin were designed as follows: forward, 5′CAACCGCGAGAAGATGACC3′ and reverse 5′ATCACGATGCCAGTGGTACG3′. The analysis was performed using the ABI StepOne Real-time qPCR system (Applied Biosystems, Foster City, CA, USA) and FastStart Universal SYBR Green Master (ROX) reagent (Roche Applied Science, Mannheim, Germany) according to the protocols. Expression was relatively normalized to the endogenous control of β-actin.

### Western Blotting Analysis

Western blotting analysis was performed on cells solubilized in RIPA buffer with 1% protease inhibitor. The solution was centrifuged at 12,000 g for 15 min at 4°C. The supernatant was obtained. Western blotting analysis was conducted with primary antibodies against PGK1 (1:500, rabbit polyclonal, Abcam, Cambridge, MA, USA), XAF1 (1:300, rabbit polyclonal, Novus Biologicals, USA), cleaved caspase-3 (1:1,000, Cell Signaling Technology, USA), Bcl-2 (1:1,000, Cell Signaling Technology, USA), and Bax (1:1,000, Cell Signaling Technology, USA). β-tubulin (1:1,000, Zhong Shan-Bio Co., Beijing, China) was used as a loading control.

### Immunohistochemistry (IHC)

A breast cancer tissue microarray was prepared for IHC as described previously (20). All breast cancer patients were routinely tested for ER, PR, and Her-2 using antibodies against the proteins of ER, PR, and Her-2 (Zhong Shan-Bio Co., Beijing, China). The samples were incubated with anti-PGK1 (1:100, rabbit polyclonal, Abcam, Cambridge, MA, USA) or anti-XAF1 antibodies (1:500, Novus Biologicals, USA) overnight followed by incubation with a rabbit secondary antibody (1:1,000, Zhong Shan-Bio Co., Beijing, China). A determination of PGK1 and XAF1 protein expression were performed by multiplying the intensity values and extension values (27). The value of four was used to differentiate between low expression and high expression. Immunohistochemical staining for ER, PR, and Her-2 was obtained as we described previously (20).

### Generation of Stable Cell Lines

**Figure 1:**
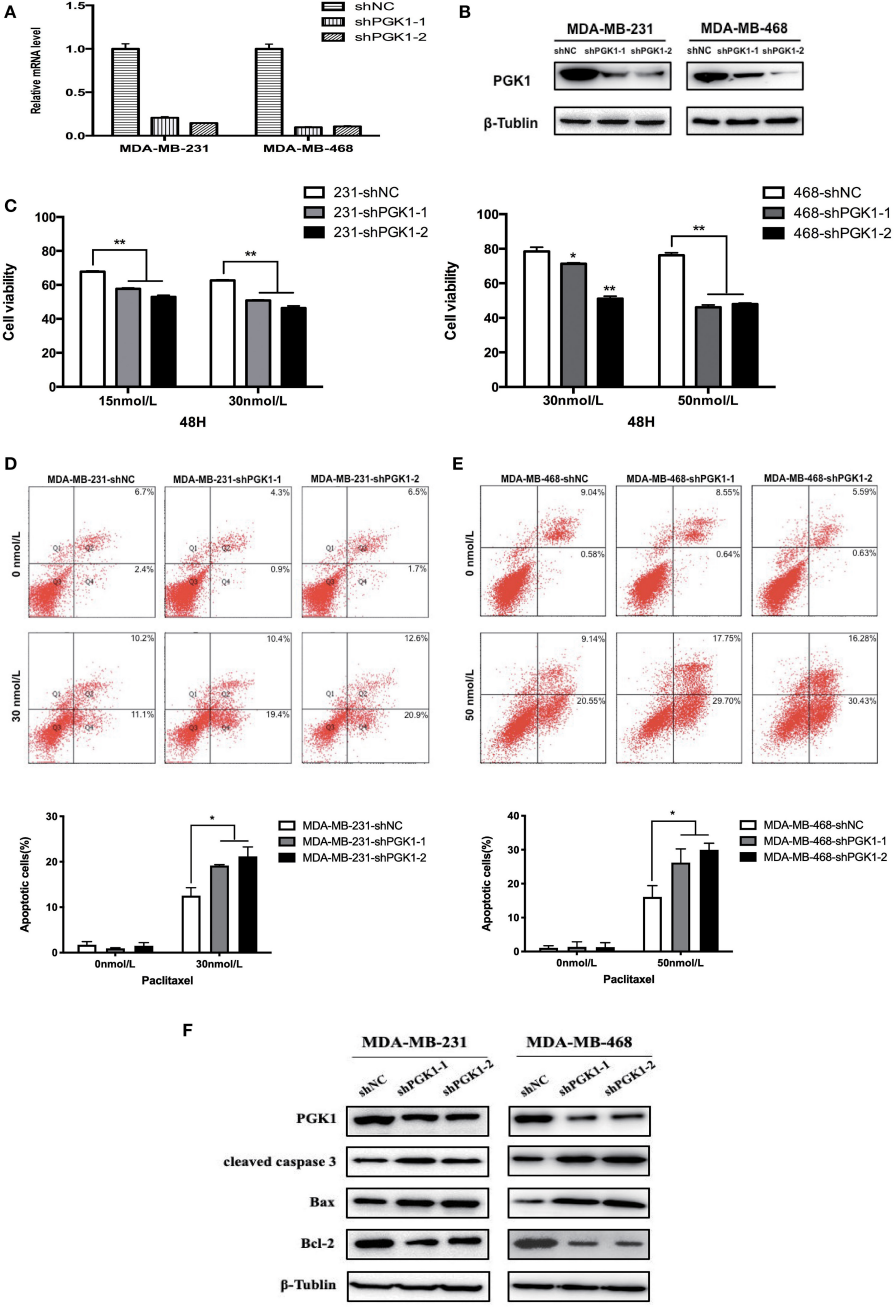
PGK1 silencing increases paclitaxel sensitivity in TNBC cell lines. **(A,B)** Real-time qPCR analysis and Western blotting analysis of PGK1 expression in TNBC cell lines transfected with vector, shPGK1-1 or shPGK1-2. **(C)** Upon treatment with paclitaxel for 48 h, a dose-dependent inhibition of cell growth was observed in PGK1-knockdown cell lines. **(D,E)** Apoptosis was evaluated by flow cytometry in PGK1-knockdown and control cell lines after treatment with paclitaxel. **(F)** Western blotting analysis of PGK1, cleaved caspase 3, Bax, Bcl-2, and β-tubulin in PGK1-silenced cell lines treated with paclitaxel. *P < 0.05,**P < 0.01.

Lentivirus constructs specific for PGK1 and the negative control were obtained from Genechem Co. (Shanghai, China). The sequence of the shPGK1 constructs were as follows: shPGK1-1, CAAGATTGTCAAAGACCTA; and shPGK1-2, TATGAAGAACAACCAGATA. The nontargeted control sequence was 5′-TTCTCCGAACGTGTCACGT-3′. Either lentivirus constructs or controls were used to infect MDA-MB-231 and MDA-MB-468 cells. The cell lines were cultured in complete media containing 2 μg/ml puromycin to generate stable knockdown cell lines 24 h after infection. Real-time qPCR and Western blotting were performed to validate the knockdown of PGK1 mRNA (Figure 1A) and protein (Figure 1B) expression, respectively.

### RNA Interference

All small interfering RNA (siRNA) constructs against XAF1 and the negative control plasmid were purchased from GenePharma (Shanghai, China). The sequences of the siRNAs used to suppress XAF1 expression were as follows: sense, 5′GCUCCUGAAAGGGAAAUCUTT3′ and antisense, 5′AGAUUUCCCUUUCAGGAGCTT3′. The non-targeted control sequences were as follows: sense, 5′UUCUCCGAACGUGUCACGUTT3′ and antisense, 5′ACGUGACACGUUCGGAGAATT3′. Cell lines were transfected with siRNA and negative control using Lipofectamine 2000 (Invitrogen, Carlsbad, CA) for 48 h according to the protocols.

### Cell Viability Assays *in vitro*

A total of 2 × 10^4^ knockdown and vector cells were suspended in 100 μl medium and seeded in 96-well plates. Paclitaxel (15 and 30 nmol/L for MDA-MB-231 cells and 30 and 50 nmol/L for MDA-MB-468 cells) was added to every well in 96-well plates. The plates were incubated for 48 h. The CellTiter 96 Aqueous One Solution Cell Proliferation Assay Kit (Promega, Madison, USA) was used according to the protocols. Then, 20 μl reagent was added to every well of the 96-well containing the cell samples and incubated in 5% CO_2_ at 37°C for 1 h. Microplate reader was used to measure the absorbance values at 490 nm.

### Flow Cytometry Cell Apoptosis Assays

The cells were concentrated and stained with a PE Annexin V Apoptosis Detection kit (BD Biosciences, USA). Then, the cells were washed twice with cold PBS. The cells were resuspended in 100 μl binding buffer. 5 μl PE Annexin V and 5 μl 7-AAD were used to stain the samples for 15 min at room temperature in the dark. 400 μl binding buffer was added after 15 min stainning. Flow cytometry assays were performed within 1 h. The assays were performed on a BD Influx Cell Sorter (BD Biosciences, San Jose, CA, USA). Results were analyzed using FACS software and calculated with the percentage of apoptotic cells (28).

### RNA Sequencing

Total RNA was extracted from MDA-MB-231-shNC, MDA-MB-231-shPGK1-1, and MDA-MB-231-shPGK1-2 cells. Then, a sequencing library of each RNA sample was prepared. BGISEQ-500 was used to profile the transcriptional analysis, which was performed by BGI Tech (Shenzhen, China). Raw sequences were aligned to the human reference genome hg38 in the UCSC genome browser. RPKM values were used for gene quantification. Gene Ontology (GO) functional enrichment analysis was performed to identify associated biological processes. Pathway analysis of differentially expressed genes was carried out to determine the important pathways based on the latest Kyoto Encyclopedia of Genes and Genomes (KEGG) database.

### Statistical Analysis

All statistical tests were conducted using Prism 7.0 (GraphPad Software, San Diego, CA, USA). Comparisons between groups for statistical significance were performed with Student's *t*-test or ANOVA. The correlation between expression of PGK1 and XAF1 in TNBC tissues was analyzed by Pearson correlation test. *P* < 0.05 indicated statistical significance.

## Results

### Downregulation of PGK1 Increases Paclitaxel Sensitivity *in vitro*

To analyze the possible role of PGK1 participate in the sensitivity of breast cancer cells to paclitaxel treatment, we used shPGK1-1 and shPGK1-2 lentiviruses to create stable PGK1-downregulated cell lines derived from the MDA-MB-231 (MDA-MB-231-shPGK1-1 and MDA-MB-231-shPGK1-2) and MDA-MB-468 (MDA-MB-468-shPGK1-1 and MDA-MB-468-shPGK1-2) cell lines. Compared with the control shRNA (MDA-MB-231-shNC and MDA-MB-468-shNC), the shPGK1 lentiviruses decreased the mRNA (Figure 1A) and protein (Figure 1B) expression of PGK1.

After treatment with 15 or 30 nmol/L paclitaxel for 48 h, a dose-dependent inhibition of cell viability was detected in MDA-MB-231-knockdown cells. PGK1-knockdown cells were more sensitive to paclitaxel treatment than vector cells. In MDA-MB-468 cells, after exposure to 30 or 50 nmol/L paclitaxel, the cell viability was lower in PGK1-knockdown cell lines than in the vector cell line significantly. Statistical analysis showed that the significant differences between PGK1-knockdown and vector cell lines were presented in both MDA-MB-231 and MDA-MB-468 cell lines (Figure 1C).

### PGK1 Plays a Significant Role in Sensitivity of Tumor Cell to Paclitaxel-Induced Apoptosis

Paclitaxel is well-known to play its cytotoxic role through the induction of apoptosis (29). Therefore, to determine whether PGK1 silencing could enhance paclitaxel-induced anticancer effects on TNBC cells, we examined apoptosis levels by flow cytometry. The number of stained cells was measured to reflect the extent of apoptosis. We observed that MDA-MB-231 PGK1-silenced cell lines were more sensitive to apoptosis which was induced by paclitaxel apoptosis than vector cells (Figure 1D). Similar results were observed in MDA-MB-468 cells, with an ~1.6~1.9-fold difference in the percentage of apoptotic cells at 48 h (Figure 1E).

These results were further confirmed by investigation of the apoptosis markers cleaved caspase-3, Bax, and Bcl-2. The results demonstrated that the expression levels of cleaved caspase-3 and Bax in MDA-MB-231 PGK1-silenced cell lines were increased compared with those in vector cells after treatment with paclitaxel for 48 h, whereas the expression levels of the anti-apoptosis protein Bcl-2 were decreased (Figure 1F). MDA-MB-468 PGK1-silenced cells manifested the same trend for apoptosis-related proteins.

### XAF1 Expression Is Significantly Increased in PGK1-Downregulated TNBC Cells

To identify potential apoptosis-related molecular mechanisms of PGK1 in TNBC, we conducted RNA transcriptional sequence analysis to investigate the differential gene expression profiles between the PGK1 downregulation groups and the vector group using the MDA-MB-231 cell line. The RNA transcriptional sequencing data is accessible with the following link: https://www.ncbi.nlm.nih.gov/sra/PRJNA665999.

**Figure 2:**
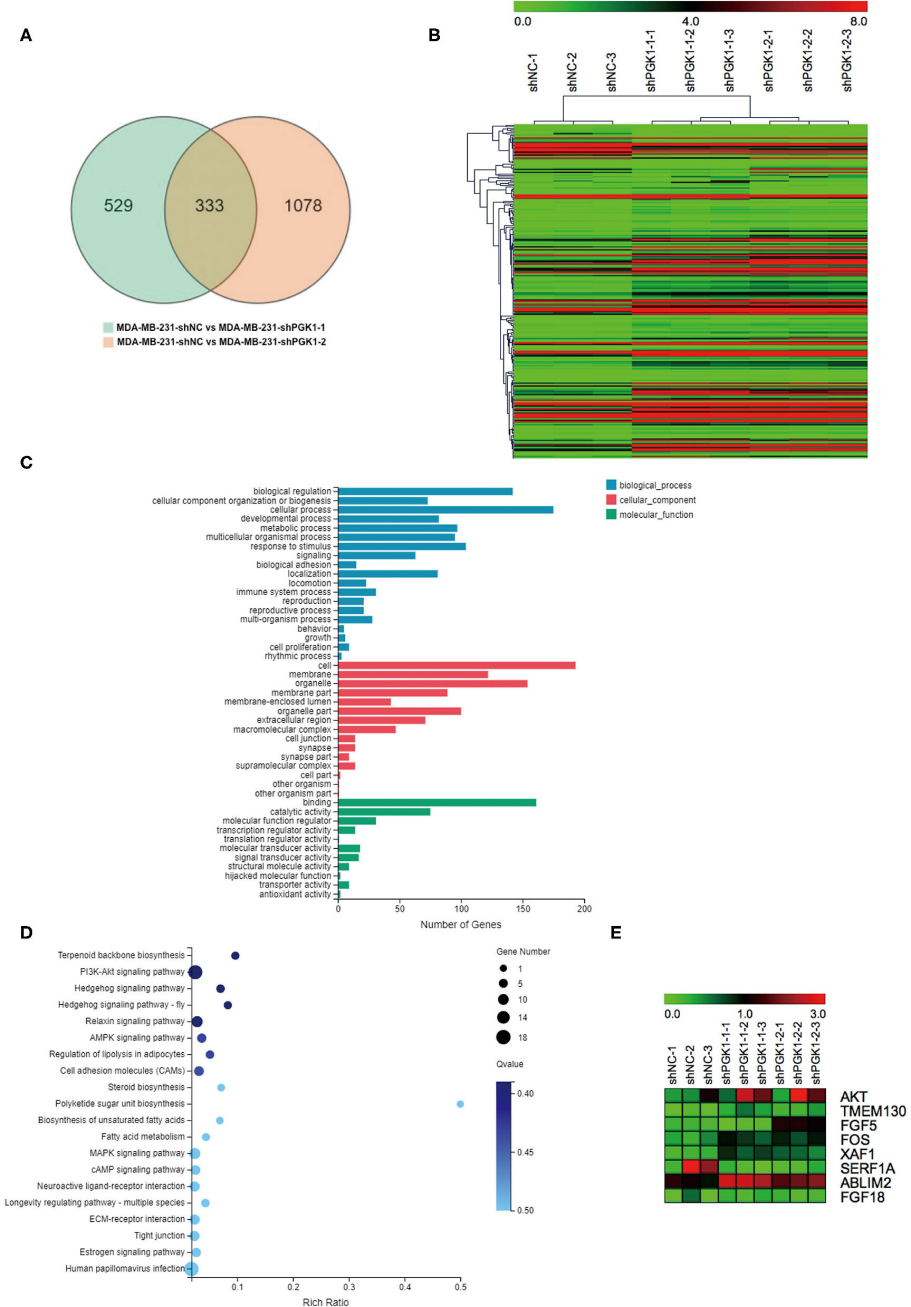
XAF1 expression level is significantly upregulated in PGK1-downregulated TNBC cell lines. **(A)** Venn diagram showing the differentially expressed genes. Numbers in the overlapped area indicate the number of differentially expressed genes. **(B)** Hierarchical clustering of quantitative gene expression profiling for MDA-MB-231-shPGK1-1, MDA-MB-231-shPGK1-2, and MDA-MB-231-shNC cells. **(C)** GO analysis of the biological processes associated with the differentially expressed genes. **(D)** KEGG pathway analysis of differentially expressed genes was carried out to determine the important pathways. **(E)** Heatmap showing the differentially expressed genes that were related to breast cancer.

By bioinformatics analysis, we found that 333 genes were differentially expressed (Figure 2A). Among the 333 differentially expressed genes, 233 were aligned to the human reference genome hg38 in the UCSC genome browser, and 100 were novel genes named by the BGI Company. All 233 UCSC-named differentially expressed genes are shown in Figure 2B. For the 233 genes, Gene Ontology (GO) analysis revealed that the genes that differentially expressed were enriched in biological progress including cellular components and molecular functions (Figure 2C).

In our present research, we found that the silencing of PGK1 by shPGK1 increased the apoptosis level induced by paclitaxel treatment in TNBC cell lines. To elucidate the potential PGK1 target genes involved in paclitaxel-induced apoptosis in TNBC, we performed KEGG pathway analysis. The analysis indicated that the 233 differentially expressed genes were enriched in 237 pathways, and the top 20 biological processes are shown in Figure 2D. Among the 237 pathways, we found that 8 genes were assigned to the breast cancer pathway (Figure 2E). Next, we focused on the selected gene set. In this group of genes, XAF1 was associated with apoptosis (30). Thus, it was chosen for analysis to measure the relationship between PGK1 and XAF1.

### PGK1 Expression and XAF1 Expression Are Negatively Correlated in TNBC Tissues

**Figure 3:**
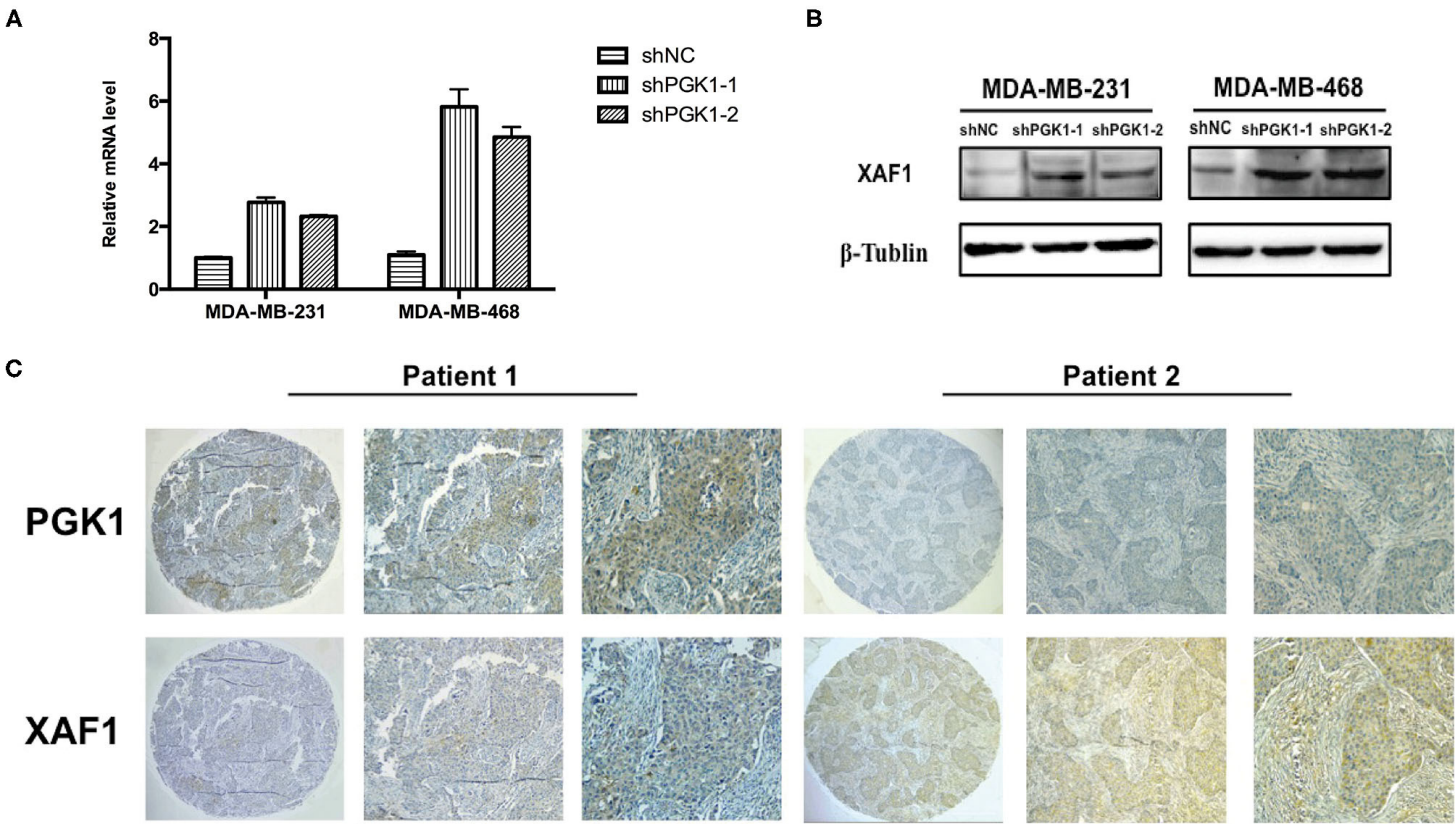
PGK1 negatively correlates with XAF1 **(A,B)** Real-time qPCR and Western blotting analysis of XAF1 and β-tubulin in PGK1-downregulated TNBC cell lines. **(C)** Immunohistochemical staining of PGK1 and XAF1 in TNBC tissues.

Real-time qPCR and Western blot analysis were used to measure XAF1 mRNA and protein expression in PGK1-downregulated cells and vector cells. We demonstrated that the level of XAF1 was greatly upregulated in PGK1-silenced TNBC cell lines (Figures 3A,B). Taken together, the results suggested a regulatory correlation between PGK1 and XAF1 in TNBC. To further validate the association of PGK1 and XAF1 expression in TNBC, an IHC assay was used to detect the expression levels of PGK1 and XAF1 in 39 TNBC patients. As shown in Figure 3C, tissues with high PGK1 expression displayed significantly lower XAF1 expression levels than tissues with low PGK1 expression. Furthermore, Pearson correlation test exhibited that there was a negative correlation between PGK1 and XAF1 expression in TNBC tissues (*P* = 0.03).

### PGK1 Regulates Paclitaxel Sensitivity Through XAF1

**Figure 4:**
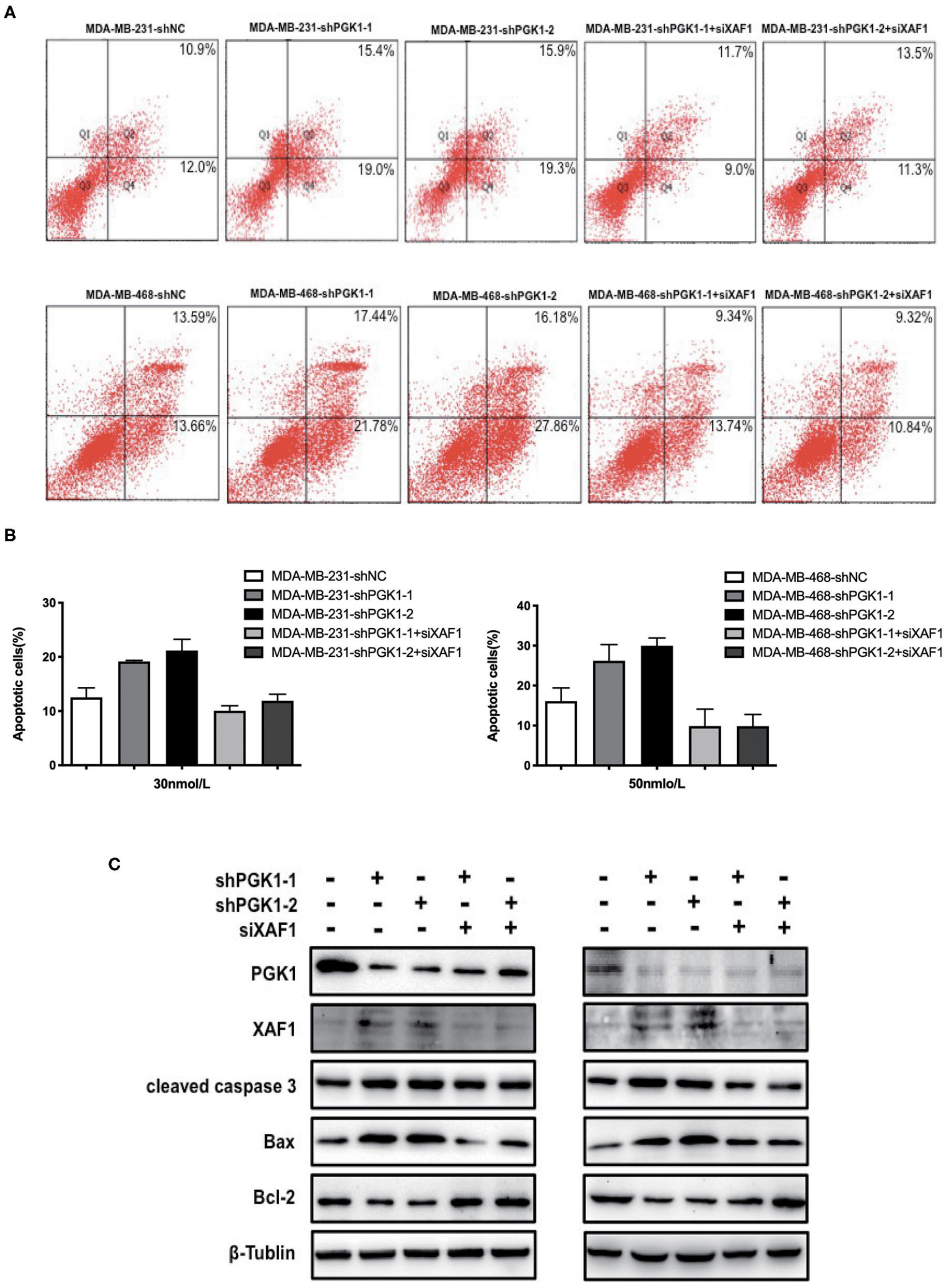
PGK1 regulates paclitaxel sensitivity through XAF1. **(A,B)** Apoptosis was evaluated by flow cytometry in PGK1-knockdown cell lines, PGK1+XAF1-silenced cell lines and control cell lines after treatment with paclitaxel. **(C)** Western blotting analysis of PGK1, XAF1, cleaved caspase 3, Bax, Bcl-2, and β-tubulin in PGK1-silenced cell lines, PGK1+XAF1-silenced cell lines and control cell lines after treatment with paclitaxel.

Because our results indicated that PGK1 could regulate XAF1 expression and that PGK1 silencing upregulated paclitaxel-treated TNBC cell motility, we hypothesized that PGK1 modulates paclitaxel sensitivity through XAF1. In order to test the hypothesis, we transduced PGK1-silenced TNBC cell lines with siXAF1 and tested the apoptosis level by flow cytometry assays in paclitaxel-treated TNBC cell lines. We observed that the paclitaxel-induced apoptosis enhancement was significantly reversed (Figure 4A). The number of stained cells was measured, and the results are shown in Figure 4B. Silencing XAF1 reduced the paclitaxel-induced apoptosis level in PGK1-knockdown cell lines. Moreover, compared with PGK1-downregulated cells, the changes in the expression levels of cleaved caspase-3, Bax and Bcl-2 were reversed in paclitaxel-treated PGK1 and XAF1-co-silenced cells (Figure 4C). Taken together, these observations indicate that PGK1 negatively correlates with XAF1 in paclitaxel sensitivity modulation.

## Discussion

High aggressiveness, as well as non-susceptibility to hormone and targeted therapies, limit the number of therapeutic opportunities and make the prognosis of TNBC patients poor. Efforts have been conducted to increase the therapeutic opportunities for TNBC patients in recent years. Recent research has shown that hypoxia-associated genes are upregulated in TNBC and are associated with resistance to chemotherapy (6). Inhibition of glycolysis is used to overcome the drug resistance of tumors (31), thus raising the probability that drugs designed to induce hypoxia may be an effective strategy for treatment of TNBC. To provide new insights into the mechanism by which glycolysis influences the tumorigenic process and treatment in TNBC, we performed the present study and showed that downregulation of the glycolysis enzyme PGK1 significantly increased the sensitivity to paclitaxel treatment in the MDA-MB-231 and MDA-MB-468 TNBC cell lines. Furthermore, we showed that downregulation of PGK1 promotes sensitivity to paclitaxel treatment by increasing the apoptosis level through upregulation of XAF1 activity.

Previously, it has been reported that upregulation of PGK1 expression is observed in several types of cancer (13, 15–18, 20). Mounting evidence has established that PGK1 plays an important role in tumorigenesis via the regulation of a variety of biological processes. PGK1 has been shown to take part in DNA replication and repair process in mammalian cell nuclei (32, 33). PGK1 functions as a glycolytic enzyme and protein kinase in the mutual regulation of metabolism and autophagy to promote tumorigenesis (34). PGK1 is highly expressed in hepatocellular carcinoma, and the silencing of PGK1 reduces cancer cell proliferation and metastasis (35).

Furthermore, PGK1 has been revealed to be activated in breast cancer. High PGK1 expression presented shorter survival in breast cancer than low PGK1 expression. The depletion of PGK1 impaired cell migration and reversed the HIF-1α-mediated epithelial-mesenchymal transition (EMT) process (36). PGK1 was also demonstrated to be a target gene that modulated cell viability, proliferation (37), invasion (38) and apoptosis (39) in breast cancer. We previously showed that elevated mRNA and protein expression levels of PGK1 were detected in breast cancer tissues compared with normal breast tissues. In addition, the increased expression of PGK1 predicted a poor outcome in breast cancer patients (20).

Paclitaxel is commonly used as the first-line chemotherapeutic drug for TNBC (40). The benefits of paclitaxel-based chemotherapy for TNBC are clear; nevertheless, response rates are low, and over half of TNBC patients develop decreased sensitivity to paclitaxel treatment by 6–10 months (41). Therefore, improvements in paclitaxel sensitivity are urgently needed. Several studies have assessed the effect of PGK1 on sensitivity and resistance to chemotherapy. High expression of PGK1 has been shown to be associated with multidrug resistance phenotypes through an MDR-1-independent mechanism (21). Inhibition of PGK1 is able to increase the vulnerability of gastric cancer cells and overcome chemotherapeutic therapy resistance (42). Zhou et al. (43) observed that PGK1 accelerates chemoresistance to cisplatin by activating HSP90/ERK pathway-mediated DNA methylation and DNA repair. Our previous study revealed a correlation between high level of PGK1 expression and poor prognosis in breast cancer. We also demonstrated that overexpression of PGK1 might be a prognostic biomarker of chemoresistance to paclitaxel treatment in breast cancer. However, the precise roles and the underlying mechanism of PGK1 in affecting paclitaxel treatment in breast cancer remain unknown, and further exploration is required.

In the present study, a cell viability assay was used to show a significant decrease in cell viability after the treatment with paclitaxel in PGK1-knockdown cell lines. In addition, we revealed that knockdown of PGK1 activated the expression of the apoptotic proteins cleaved caspase-3 and Bax and inhibited the expression of the anti-apoptotic protein Bcl-2 in paclitaxel-treated TNBC cell lines. Therefore, we propose that downregulation of PGK1 may be involved in apoptosis-mediated paclitaxel sensitivity in TNBC.

A promising approach, which was used to enhance the effectiveness of chemotherapy drugs or to induce apoptotic cell death, is to target the apoptotic pathway directly. On the basis of this idea, we examined the effects of PGK1 depletion on genes involved in breast cancer. RNA sequencing was used to assess the differentially expressed genes and the enrichment of functional pathways. The analysis identified 233 differentially expressed genes. Eight of the genes were involved in the breast cancer pathway. Among the 8 genes, XAF1 was upregulated by PGK1 knockdown. Upregulation of XAF1 expression was observed to suppress cancer cell growth and increase cell sensitivity to chemotherapy (30).

XAF1 was first identified as a XIAP-interacting protein to suppress the anti-caspase activity of XIAP1 (44). It has been shown that XAF1 is expressed in normal cells and tissues but present at very low or undetectable levels in tumor cell lines and tissues as a tumor suppressor (45, 46). Moreover, upregulation of XAF1 expression increases cell sensitivity to chemotherapy (30, 47). S.K. Chung et al. suggested that XAF1 expression suppressed tumor cell growth, led to caspase-3 activation and enhanced the apoptotic sensitivity of tumor cells to various apoptotic stimuli in colorectal tumors, while siRNA-mediated knockdown of XAF1 greatly enhanced cellular survival under apoptotic stresses (30). Restoration of XAF1 expression significantly increased apoptosis level in gastric cancer cell lines and improved the sensitivity of gastric cancer cells to 5-fluorouracil and cisplatin-induced apoptosis (47).

In the present work, we verified the relationship between PGK1 and XAF1 expression for the first time. Immunohistochemical analysis of TNBC patient tissues confirmed the negative correlation between PGK1 and XAF1. In addition, increased expression of XAF1 occurred in parallel with increased apoptosis in PGK1-knockdown cells treated with paclitaxel. We hypothesize that depletion of PGK1 improves the sensitivity of TNBC cells to paclitaxel treatment through increased XAF1 expression. We transduced PGK1-silenced TNBC cell lines with siXAF1 and examined the apoptosis level by flow cytometry assays and apoptosis-associated proteins by Western blotting analysis in paclitaxel-treated TNBC cell lines. We observed that the paclitaxel-induced apoptosis enhancement was significantly reversed. Therefore, the paclitaxel-induced apoptosis enhanced by the silencing of PGK1 required XAF1 upregulation in TNBC.

In conclusion, our results demonstrate that PGK1 plays an important role in paclitaxel-induced apoptosis. Silencing of PGK1 confers sensitivity to paclitaxel *in vitro* mediated via XAF1 expression in TNBC. However, several other issues also remain to be addressed, and more studies are required to analyze the precise role of PGK1. It would be of great interest to know whether other pathways are also involved in mediating the anti-apoptotic effect of PGK1 in TNBC. Thus, on the basis of our present study, we provided new insight into the role of PGK1 in TNBC. PGK1 might be a novel therapeutic target in TNBC and has important implications in the development of targeted therapeutics for improving paclitaxel sensitivity in patients receiving paclitaxel-based chemotherapy.

## Data Availability Statement

The datasets presented in this study can be found in online repositories. The names of the repository/repositories and accession number(s) can be found at: the NCBI Sequence Read Archive, ID: PRJNA665999, (https://www.ncbi.nlm.nih.gov/sra/PRJNA665999).

## Ethics Statement

The studies involving human participants were reviewed and approved by Harbin Medical University. The patients/participants provided their written informed consent to participate in this study.

## Author Contributions

SS and DP designed the research. HW, XW, ZY, YJ, WW, YS, YZ, and ZC performed the research. SS, YJ, YC, and XL analyzed the data. SS drafted the manuscript and wrote the paper. All authors approved the manuscript.

## Conflict of Interest

The authors declare that the research was conducted in the absence of any commercial or financial relationships that could be construed as a potential conflict of interest.
